# Protective Effects of a Discontinuous Treatment with Alpha-Lipoic Acid in Obesity-Related Heart Failure with Preserved Ejection Fraction, in Rats

**DOI:** 10.3390/antiox9111073

**Published:** 2020-10-31

**Authors:** Cristina Pop, Maria-Georgia Ștefan, Dana-Maria Muntean, Laurențiu Stoicescu, Adrian Florin Gal, Béla Kiss, Claudiu Morgovan, Felicia Loghin, Luc Rochette, Benjamin Lauzier, Cristina Mogoșan, Steliana Ghibu

**Affiliations:** 1 Department of Pharmacology, Physiology and Pathophysiology, Faculty of Pharmacy, “Iuliu Haţieganu” University of Medicine and Pharmacy, 400349 Cluj-Napoca, Romania; pop.cristina@umfcluj.ro (C.P.); cmogosan@umfcluj.ro (C.M.); steliana.ghibu@umfcluj.ro (S.G.); 2 Department of Toxicology, Faculty of Pharmacy, “Iuliu Haţieganu” University of Medicine and Pharmacy, 400349 Cluj-Napoca, Romania; stefan.georgia@umfcluj.ro (M.-G.Ş.); floghin@umfcluj.ro (F.L.); 3 Department of Pharmaceutical Technology and Biopharmaceutics, Faculty of Pharmacy, “Iuliu Haţieganu” University of Medicine and Pharmacy, 400012 Cluj-Napoca, Romania; dana.muntean@umfcluj.ro; 4 Department of Cardiology, Vth Medical Clinic, Faculty of Medicine, “Iuliu Haţieganu” University of Medicine and Pharmacy, 400139 Cluj-Napoca, Romania; 5 Department of Cell Biology, Histology and Embryology, Faculty of Veterinary Medicine, University of Agricultural Sciences and Veterinary Medicine, 400372 Cluj-Napoca, Romania; adrian.gal@usamvcluj.ro; 6 Preclinical Department, Faculty of Medicine, “Lucian Blaga” University of Sibiu, 550169 Sibiu, Romania; claudiu.morgovan@ulbsibiu.ro; 7 Equipe d’Accueil (EA 7460), Physiopathologie et Epidémiologie Cérébro-Cardiovasculaires (PEC2), Université de Bourgogne-Franche Comté, Faculté des Sciences de Santé, 7 Bd Jeanne d’Arc, 21000 Dijon, France; luc.rochette@u-bourgogne.fr; 8 Université de Nantes, CHU Nantes, CNRS, INSERM, L’institut du Thorax, F-44000 Nantes, France; benjamin.lauzier@univ-nantes.fr

**Keywords:** heart failure with preserved ejection fraction, obesity, oxidative stress, antioxidants, alpha-lipoic acid

## Abstract

Obesity induces hemodynamic and humoral changes that are associated with functional and structural cardiac remodeling, which ultimately result in the development of heart failure (HF) with preserved ejection fraction (HFpEF). In recent years, pharmacological studies in patients with HFpEF were mostly unsatisfactory. In these conditions, alternative new therapeutic approaches are necessary. The aim of our study was (1) to assess the effects of obesity on heart function in an experimental model and (2) to evaluate the efficacy of an alpha-lipoic acid (ALA) antioxidant treatment. Sprague-Dawley rats (7 weeks old) were either included in the control group (*n* = 6) or subjected to abdominal aortic banding (AAB) and divided into three subgroups, depending on their diet: standard (AAB + SD, *n* = 8), hypecaloric (AAB + HD, *n* = 8) and hypecaloric with discontinuous ALA treatment (AAB + HD + ALA, *n* = 9). Body weight (BW), glycemia, echocardiography parameters and plasma hydroperoxides were monitored throughout the study. After 36 weeks, plasma adiposity (leptin and adiponectin) and inflammation (IL-6 and TNF-alpha) markers, together with B-type natriuretic peptide and oxidative stress markers (end-products of lipid peroxidation and endogenous antioxidant systems) were assessed. Moreover, cardiac fiber diameters were measured. In our experiment, diet-induced obesity generated cardiometabolic disturbances, and in association with pressure-overload induced by AAB, it precipitated the onset of heart failure, cardiac hypertrophy and diastolic dysfunction, while producing a pro-oxidant and pro-inflammatory plasmatic status. In relationship with its antioxidant effects, the chronic ALA-discontinuous treatment prevented BW gain and decreased metabolic and cardiac perturbations, confirming its protective effects on the cardiovascular system.

## 1. Introduction

Heart failure (HF) is a clinical syndrome associated with a significant risk of mortality, hospitalization and impairment of quality of life [1]. Approximately half of all HF patients are currently diagnosed with HF with preserved ejection fraction (HFpEF) and it is estimated that its prevalence will soon exceed that of the other HF subtypes [2,3], especially due to population aging, the increased global incidence of obesity, as well as metabolic and cardiovascular disorders derived from excess adipose tissue [2,4]. HFpEF is mainly characterized by signs and symptoms typical for HF (dyspnea, fatigue, intolerance to effort, peripheral edema, etc.), normal or mildly reduced ejection fraction (EF ≥ 50%), left ventricular (LV) hypertrophy, left atrial enlargement and/or diastolic dysfunction [1,5,6]. Moreover, HFpEF is a multifactorial disease; most patients are elderly and most often women, with a history of hypertension, obesity, diabetes mellitus, hyperlipidemia, atrial fibrillation or renal dysfunctions, while heart failure with reduced ejection fraction (HFrEF) develops more frequently in men with a history of myocardial ischemic events and myocardial infarction [1,5,6,7,8]. Obesity represents a major non-cardiac comorbidity of HFpEF and obesity-related HFpEF constitutes a distinct clinical phenotype [9,10] with a particular biomarker profile characterized by overproduction of adipocyte-derived signaling molecules such as leptin and neprilysin, and the consequences thereof (i.e., hypervolemia and lower levels of natriuretic peptides) [11]. Studies suggest that the main link between comorbidities and HFpEF seems to be a systemic pro-inflammatory state [12,13,14] with multiple triggers that subsequently affects coronary microvascular endothelium function, cardiomyocyte structure and heart function [12,13].

In overweight and obese persons, the first step in the development of a systemic pro-inflammatory state is represented by monocyte infiltration in adipose tissue (especially in visceral adiposity). The recruitment of monocytes is initiated by the high plasma saturated fatty acids (SFA) levels that bind to Toll-like receptors on the surface of adipocytes or resident immune cells and stimulate synthesis of monocyte chemoattractant protein-1 (MCP-1/CCL2) and pro-inflammatory cytokines (TNF-alpha, IL-6, etc.). MCP-1/CCL2 is the key element responsible for the chemotaxis of monocytes and the circulating leptin facilitates their diapedesis. Additionally, during adipose tissue expansion, tissue hypoxia and adipocyte death enhance monocyte infiltration. The newly formed tissue macrophages exhibit a pro-inflammatory profile and amplify the inflammatory process [15,16]. Subsequently, in a second step, other pathophysiological events take place, such as subendothelial circulating-leukocyte migration and the increase in reactive oxygen species (ROS) synthesis by the coronary microvascular endothelial cells [2]. All of these processes lead to endothelial dysfunction and limit nitric oxide (NO) bioavailability to adjacent cardiomyocytes. Low NO bioavailability decreases protein kinase G (PKG) activity in cardiomyocytes and titin phosphorylation via NO-sGC-cGMP-PKG signaling downregulation. The final step is represented by LV remodeling (concentric hypertrophy) with LV relaxation impairment [8]. Cardiomyocyte hypertrophy occurs as a consequence of the impairment of PKG-mediated antihypertrophic activity and at the same time, differentiated myofibroblasts stimulate interstitial collagen I deposition, a process mediated by subendothelial leukocyte infiltration and profibrotic cytokines (TGFβ–transforming growth factor β) [2,12,13]. Both processes increase cardiomyocyte stiffness and interstitial fibrosis with impairment of myocardium relaxation and development of diastolic LV dysfunction [2,8,12].

Additionally, metabolic and cardiovascular disorders associated with obesity are characterized by significant increases in ROS production and the breakdown of endogenous antioxidant systems. This creates a vicious circle which amplifies the pre-existing disturbances [17,18,19,20,21].

The presence of significant oxidative stress (OS) that impairs cardiomyocyte NO bioavailability and general endothelial function encourages the evaluation of antioxidant efficacy in HFpEF. However, the selection of an appropriate antioxidant for a particular pathophysiological context can present a real challenge. The redox perturbations involved in HFpEF are multiple and not fully understood. In this context, the ideal antioxidant should, besides having a good safety profile, reach the target tissues and influence multiple signaling pathways. In this field, alpha-lipoic acid (ALA) could be a promising candidate as it acts via a complex antioxidant mechanism, being able to scavenge reactive oxygen species (HO·, HClO, ^1^O_2_), chelate divalent transition metals and regenerate the reduced form of some endogenous antioxidants: glutathione, vitamins C and E. Moreover, due to its high redox potential (–320 mV), the ALA/dihydrolipoic acid (DHLA) system offers more protection from oxidative damage; it could be even more effective than the endogenous reduced/oxidized glutathione system [22]. Moreover, its safety profile is well characterized [23] and its specific amphiphilic properties could allow for a better intra-cellular and extra-cellular distribution [22]. Consequently, multiple studies have reported the benefits of ALA treatment in metabolic syndrome (hyperglycemia, tissue insulin resistance and dyslipidemia) [17], hypertension [17,24] and obesity [25,26,27].

In this context, the aim of our study was to assess (1) the cardiac repercussions of overweight/obesity and (2) the impact of a long-term discontinuous treatment with ALA, in an animal model associating cardiac pressure-overload with diet-induced obesity, two important risk factors underlying HFpEF development.

## 2. Materials and Methods

### 2.1. Animals and Experimental Protocol

This study was approved by the Ethics Committee of the “Iuliu Hatieganu” University of Medicine and Pharmacy, Cluj-Napoca, Romania (no. 237/31.05.2018) and was performed in accordance with EU Directive 2010/63/EU for animal experiments.

Sprague-Dawley rats were purchased from “Cantacuzino” National Medico-Military Institute for Research and Development, Bucharest, Romania. Rats were housed 3–4 per cage in standard laboratory conditions (temperature 22 ± 2 °C, 12 h light/dark cycles) and received water and standard pellet food ad libitum. After one week of accommodation, at the age of 7 weeks, rats were subjected to abdominal aortic banding (AAB, *n* = 25), as previously described [28,29] or to sham operation (Control group, *n* = 6). In brief, after anesthesia (1 mL/kg dose of ketamine/xylazine/diazepam 1:1:1, intramuscularly administered), the descending aorta was detached from the vena cava and ligatured above the renal artery bifurcation using a suture thread (4–0 silk) and a 22G (0.7 mm diameter) blunt needle. Sham operated rats underwent a similar procedure, but without AAB. In order to prevent infections, a dose of 10 mg enrofloxacin/1 mL NaCl 0.09% was subcutaneously administered.

**Figure 1:**
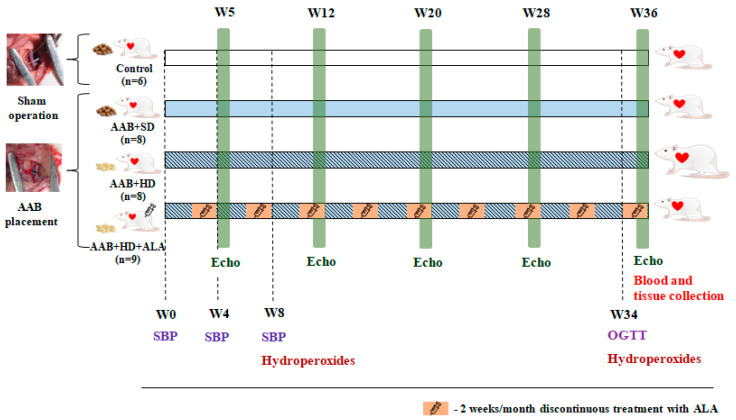
Experimental protocol. AAB—abdominal aortic banding, W—week, Echo—cardiovascular echography, SBP—systolic blood pressure, OGTT—oral glucose tolerance test, ALA—alpha-lipoic acid.

Two weeks after surgery, rats subjected to AAB were further divided into three groups depending on the type of diet used and treatment applied (Figure 1), as follows:

- AAB group fed with standard diet (AAB + SD, *n* = 8) included rats subjected to AAB, fed with standard diet during the study and injected intraperitoneally (i.p.) with saline solution (NaCl 0.09%).
- AAB group fed with hypercaloric diet (AAB + HD, *n* = 8) included rats subjected to AAB, fed with hypercaloric diet (24% fat and 11% fructose) and injected (i.p.) with saline solution (NaCl 0.09%).
- AAB group fed with hypercaloric diet and treated with ALA (AAB + HD + ALA, *n* = 9) included rats subjected to AAB, fed with hypercaloric diet and injected (i.p.) with 50 mg/kg/day ALA (Acid Tioctic^®^ 600 mg/24 mL, Rompharm, Romania) for 2 weeks/month over a period of 36 weeks (9 months).
- Control group included sham operated rats, fed with standard diet and injected (i.p.) with saline solution.

Each group of rats was injected (i.p.) with saline solution (NaCl 0.09%) or ALA for 2 weeks/month (W3-W4, W7-W8, W11-W12, W15-W16, W19-W20, W23-W24, W27-W28, W31-W32 and W35-W36) over a period of 36 weeks (9 months).

The specific food for rats, namely standard diet (SD; 13% kilocalories from fat) and hypercaloric diet (HD; 46% kilocalories from fat), was purchased from Ssniff, Soest, Germany. The hypercaloric diet was composed of 24% crude fat, 22.3% protein, 22.9% sugar, 11% fructose, 6.8% starch, 9% dextrin and 4% vitamin and mineral premix.

Body weight of rats was monitored weekly during the 36 weeks of study. In addition, food consumption was measured every three days. After 36 weeks from the beginning of the study, the rats were fasted overnight and were anesthetized with a mixture of ketamine/xylazine 1:2 (*v*/*v*), administered i.m. Blood was collected from the retro-orbital sinus, heart and abdominal adipose tissue were harvested, weighed and processed for histological analysis. Serum and plasma (from EDTA-treated blood) were separated by centrifugation and the aliquots were stored at −80 °C until analyses were performed.

### 2.2. Systolic Blood Pressure

Systolic blood pressure (SBP) was measured in conscious rats every 4 weeks by a non-invasive tail-cuff method (58,500-Ugo Basile blood pressure recorder coupled with a rodent heater, Hugo Basile, Italy) during the first 8 weeks of the study. The mean of six consecutive readings was recorded as the individual blood pressure value. Starting with week 12 (W12), due to the low signal recorded from tail photoelectric sensor and to the increased risk of exitus for the operated animals (during SBP measurement the rats were immobilized in restrainers and pre-warmed at 30 °C), systolic blood pressure monitoring was stopped.

### 2.3. Cardiovascular Echography

Heart structure and function were evaluated by echocardiography at 5, 12, 20, 28 and 36 weeks of the study. About 15–20 min after general anesthesia, rats were placed in the dorsolateral position and transthoracic echocardiography was performed using a commercially available echocardiograph equipped with a 7.5 MHz electric transducer (Ultrasonix, Boston, MA, USA). Measurements were performed using the leading-edge method of the American Society of Echocardiography [30].

Left ventricular (LV) diastolic wall thickness (interventricular septum (IVS) and posterior wall (PW)) along with LV diameters and volumes during diastole and systole were measured in parasternal long axis view using M-mode images at the level of the papillary muscles. Relative wall thickness (RWT) was calculated as RWT = (2 × PWd)/LVIDd; LVID-LV internal diameter, d-in diastole). LV mass index was calculated as LV mass index = LV mass/LV end-diastolic volume [31]. Moreover, LV ejection fraction (EF) was calculated automatically (Simpson’s method) based on echocardiographic measurements determined from a B-mode apical 4-chamber view.

Transmitral inflow was assessed by using pulsed-wave Doppler in an apical 4-chamber view (or in apical 3 chamber view when apical 4-chamber view was not available) placing the sample volume at the tip of the mitral valves.

The Doppler indexes included the peak velocity of early (E) and late (A) filling of mitral inflow and the ratio of peak velocity of early-to-late filling of mitral inflow (E/A). Moreover, using TDI (tissue Doppler imaging) we assessed early diastolic myocardial relaxation and active atrial contraction in late diastole (e′ and a′). TDI velocity measurements are obtained by placing the sample volume at the mitral annular level. Mitral valve deceleration time (MVdec), isovolumic relaxation and contraction times (IVRT and IVCT) and mitral valve ejection time (ET) were also measured. Moreover, a myocardial performance index was calculated as Tei index = (IVRT + IVCT)/ET [32].

To confirm the presence of the ligature at the abdominal aorta level and to evaluate the degree of aortic stenosis obtained, B-mode images were acquired at the same time-points as heart measurements.

All measurements were performed by the same investigator and were quantified and averaged over two consecutive cardiac cycles.

### 2.4. Blood/Plasma Measurements

#### 2.4.1. Biochemical Investigations

The fasting glycemia (mg/dL) was measured in tail vein blood, every 9 weeks, by using a glucometer (Accu-Chek, Roche, Mannheim, Germany). Furthermore, oral glucose tolerance test (OGTT) was performed at week 34 of the study, by using the same method, after overnight fasting glycemia (12 h) had been determined. Subsequently, rats were given by gavage a glucose load of 2 g/kg body weight from a 40% glucose solution, and glycemia was measured at 30, 60, 90, and 120 min after oral glucose administration [33]. For each measurement, the mean of three consecutive readings was recorded as the individual glycemia value.

Other serum biochemical markers such as plasma lipoproteins (total cholesterol, low-density lipoprotein cholesterol, high-density lipoprotein cholesterol and triglycerides), uric acid, C-reactive protein (CRP) levels, and alanine aminotransferase (ALAT) and aspartate aminotransferase (ASAT) activities were determined by spectrophotometric methods, using commercially available kits from Thermo SCIENTIFIC (Vantaa, Finland) and a Konelab 20i automatic analyzer (Thermo SCIENTIFIC, Vantaa, Finland).

#### 2.4.2. Adiposity Markers

Plasma leptin was measured with a RayBiotech (RayBiotech, Norcross, GA, SUA) ELISA kit (intra-assay coefficient of variability (CV): <10%; inter-assay CV: <12%) and plasma adiponectin (ADP/Acrp30) with an Elabscience (Elabscience, Houston, Texas, TX, SUA) ELISA kit (intra-assay CV: 5.20–6.33%; inter-assay CV: 4.09–6.93%), according to the manufacturer’s instructions.

#### 2.4.3. Inflammatory Markers

Circulating pro-inflammatory cytokines were determined in undiluted plasma by enzyme-linked immunosorbent assay (ELISA), according to the manufacturer’s instructions. Plasma interleukin-6 (IL-6) was measured using an Elabscience (Elabscience, Houston, Texas, SUA) ELISA kit (intra-assay CV: 4.83–5.90%; inter-assay CV: 4.90–6.93%) and for tumor necrosis factor-α (TNF-α) a BOSTER (BOSTER Biological Technology, Pleasanton, CA, SUA) ELISA kit (intra-assay CV: 4.1–7.7%; inter-assay CV: 6.0–8.1%) was used.

#### 2.4.4. Brain Natriuretic Peptide

Plasma concentration of brain natriuretic peptide (BNP) was determined by using a RayBiotech EIA kit (RayBiotech, Norcross, GA, SUA) (intra-assay CV: <10%; inter-assay CV: <15%), according to the manufacturer’s instructions.

#### 2.4.5. Angiotensin II

Plasma Angiotensin II (Ag II) was measured with a RayBiotech (RayBiotech, Norcross, GA, USA) EIA kit (intra-assay CV: <10%; inter-assay CV: <15%), according to the manufacturer’s instructions.

#### 2.4.6. Oxidative Stress Markers

##### Plasma Lipid Peroxidation

Plasma hydroperoxides were measured at 8 and 34 weeks of the study, by the “FORT” colorimetric test (Callegari, Parma, Italy) [34,35].

Plasma malondialdehyde (MDA, µmol/L), the end-product of lipid peroxidation was assessed by a liquid chromatographic method with UV detection (307 nm) [36].

##### Circulating Antioxidants Levels

Reduced glutathione (GSH) was determined in plasma after deproteinization with 10% metaphosphoric acid (*m*/*v*), followed by derivatization with ortho-phthalaldehyde (OPA) in the presence of 0.1% EDTANa_2_ solution in phosphate buffer (Na_2_HPO_4_ 0.1M, pH = 8) at room temperature. For oxidized glutathione (GSSG) analysis, the deproteinization was followed by incubation with N-ethylmaleimide and derivatization with OPA in the presence of 0.1M NaOH. The chromatographic analysis was performed using a Waters Acquity UPLC system coupled with Waters Acquity fluorescence detector (Waters, Milford, MA, USA), using λexc = 350 nm and λem = 420 nm, respectively. The separation was achieved by using an HSS T3 Acquity UPLC column (1.8 µm, 2.1 × 100 mm) for both glutathione forms, with isocratic elution for GSH and gradient elution for GSSG, respectively.

The catalytic activity of circulating catalase was assessed via the method described by Aebi [37]. A catalytic unit decomposes 1 µmol H_2_O_2_/minute, at pH = 7 and at 25 °C. The decomposition rate of H_2_O_2_ was monitored by observing the decrease in absorbance at 240 nm/minute.

The Total Antioxidant Capacity (TAC) was assessed by a spectrophotometric method described by Erel [38]. For the calibration curve, 6-hydroxy-2,5,7,8-tetramethylchroman-2-carboxylic acid (Trolox) was used and the results are expressed in mmol Trolox equivalent/L.

#### 2.4.7. Plasma Nitrite and Nitrate (NO_x_) Levels

Total plasma NO_x_ were assayed as the sum of plasma nitrite and nitrate, by a slightly modified method [39]. In brief, plasma nitrate was reduced to nitrite, using vanadium chloride, under acidic conditions, at 37 °C, in a 96 well microtiter plate. The newly obtained nitrite, together with pre-existent plasma nitrite were determined spectrophotometrically, using the Griess reaction (diazotization of sulfanilic acid, followed by coupling with alpha-naphthylamine) [40].

### 2.5. Histological Analysis

The cardiac tissue was collected in all rats from the same region, i.e., a transverse slice from the lower third of the heart (0.5 cm tissue sections). For histological assessment of the heart, the harvested tissue samples were fixed in 10% neutral buffered formalin and were later embedded in paraffin. The paraffin-embedded samples were sectioned at 4 μm in thickness with the Leica rotary microtome (RM2125, Nussloch, Germany) and stained with hematoxylin and eosin (H&E). The cardiac microphotographs were acquired using an OlympusBX51 microscope (Tokyo, Japan) connected to a digital camera (Olympus DP-25, Tokyo, Japan).

The diameter of cardiac muscle fibers (short-axis diameter) was measured semi-automatically with the aid of the Olympus system for image acquisition and analysis (i.e., Olympus Cell B software, Tokyo, Japan). For each assessed rat, 10 randomly selected cardiac fibers were measured and averaged in highly magnified microphotographs (i.e., objective magnification degree 40×). In each case, diameter measurements were performed in the middle third of the myocardium wall.

### 2.6. Statistical Analysis

All data are expressed as mean ± SEM (standard error of mean). To compare the four groups at a certain time or at the end of the study, statistical analyses were performed with the one-factor analysis of variance (ANOVA) test. To compare the evolution of body weight, glycemia and systolic blood pressure throughout the period of the study, two-factor repeated measures ANOVA test was used. All statistical analyses were performed using the SigmaStat software, version 4. Significance was established at a value of *p* < 0.05.

## 3. Results

### 3.1. Body Weight and Nutritional Profile

**Figure 2:**
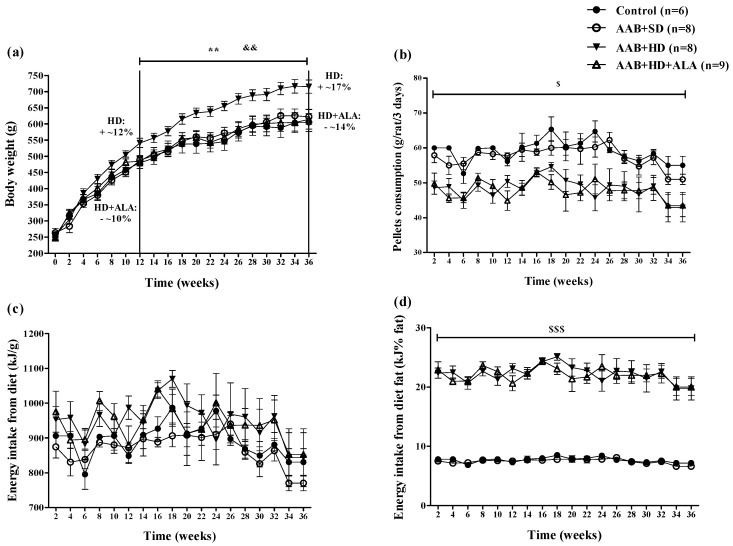
Evolution of (**a**) Body weight, (**b**) Pellets consumption (g/rat/3 days), (**c**) Energy intake from diet (kJ/g) and (**d**) Energy intake from diet fat (kJ% fat) in the 4 groups of rats evaluated. ** *p* < 0.01: AAB + HD vs. AAB + SD and Control groups; ^&&^ *p* < 0.01: AAB + HD + ALA vs. AAB + HD; ^$^ *p* < 0.05, ^$$$^ *p* < 0.001: AAB + HD and AAB + HD + ALA vs. Control and AAB + SD (two-factor repeated measures ANOVA).

Body weight (BW) increased in all four groups over the 36 weeks (W36) of the study. However, starting with week 12 (W12), hypercaloric diet (HD) induced an increase (*p* < 0.01) in BW with ~12% over the standard diet fed groups (Control and AAB + SD), a trend that was maintained until the end of the study (Figure 2a). Moreover, starting at W12, it was observed that the chronic discontinuous treatment with ALA prevented weight gain in HD fed rats, since the AAB + HD + ALA group showed body weights 10% lower (*p* < 0.01) than those of the AAB + HD group. The maximum effect was observed at W36, when the difference in body weight between AAB + HD + ALA and AAB + HD groups was ~14% (Figure 2a).

Concerning food pellets consumption throughout the study, animals fed with hypercaloric diet (AAB + HD and AAB + HD + ALA groups) consumed a significantly (*p* < 0.05) lower quantity of food pellets (grams) compared to those fed with standard diet (AAB + SD and Control groups) (Figure 2b). Although the total energy intake from diet did not differ between groups (Figure 2c), the energy intake from dietary fat was significantly (*p* < 0.001) higher in the HD fed groups (Figure 2d). Additionally, in the AAB group fed with HD and concomitantly treated with ALA (AAB + HD + ALA group), no difference in pellet consumption and energy intake from dietary fat was observed compared to the AAB + HD group (Figure 2b,d).

### 3.2. Systolic Blood Pressure

**Figure 3:**
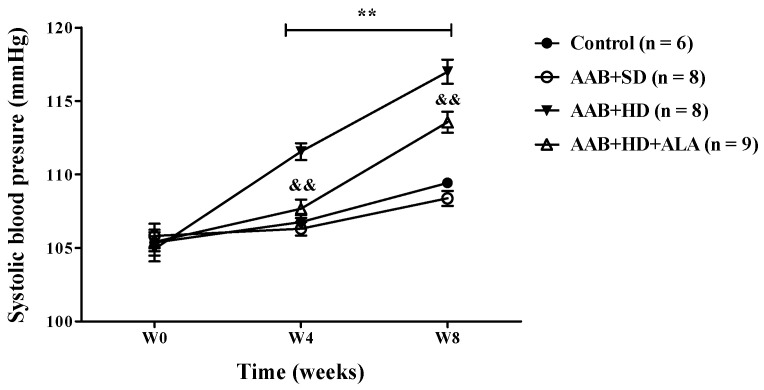
Evolution of systolic blood pressure (SBP) during the first 8 weeks of the study. ** *p* < 0.01: AAB + HD vs. AAB + SD and Control groups; ^&&^ *p* < 0.01: AAB + HD + ALA vs. AAB + HD (two-factor repeated measures ANOVA).

In ligatured rats, only the hypercaloric diet (AAB + HD group) induced an increase (*p* < 0.01) in systolic blood pressure (SBP) at 4 and 8 weeks of the study (the period when SBP could be recorded). An attenuation of this increase in SBP was observed in ALA-treated rats, since W4 of the study (Figure 3). No differences in SBP values were observed between ligatured rats fed with standard diet (AAB + SD group) and the Control group (Figure 3).

### 3.3. Cardiovascular Echography

**Figure 4:**
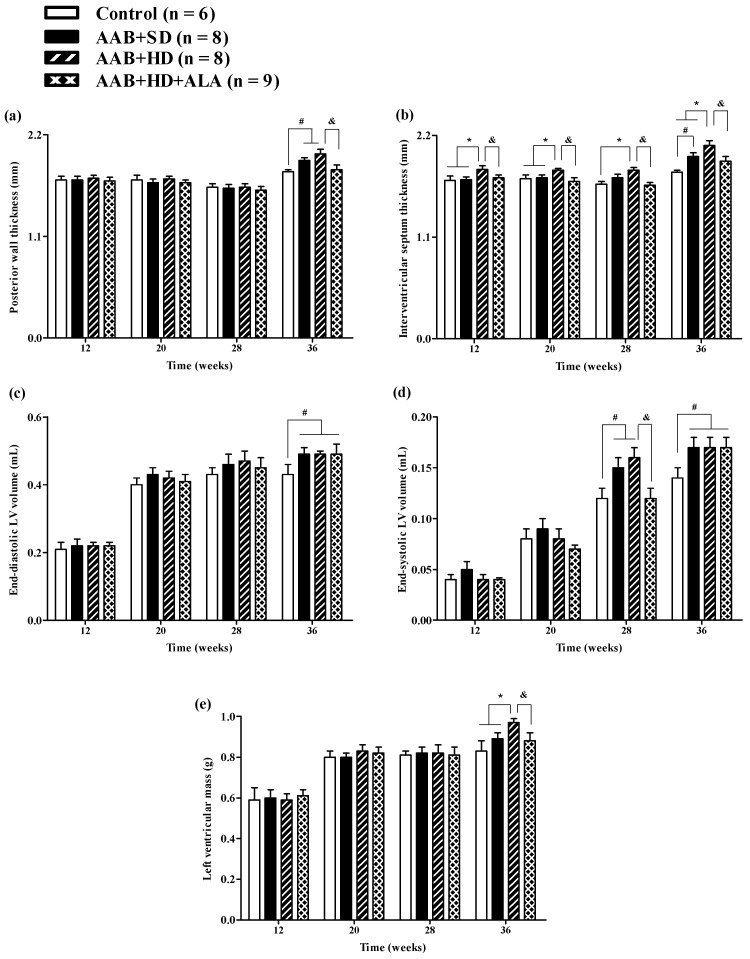
Evolution of structural echocardiographic parameters at 12-, 20-, 28- and 36-weeks of the study: (**a**) Posterior wall thickness; (**b**) Interventricular septum thickness; (**c**) End-diastolic LV volume; (**d**) End-systolic LV volume; (**e**) LV mass. ^#^ *p* < 0.05: vs. Control, * *p* < 0.05: vs. AAB + SD and Control; ^&^ *p* < 0.05: vs. AAB + HD (one-factor ANOVA).

Structural analysis of the heart, performed by echocardiography revealed that at W36 a significant (*p* < 0.05) increase in both LV posterior wall (PW) and interventricular septum (IVS) thickness was present in both ligatured groups (AAB + SD and AAB + HD) regardless of the type of food used (Figure 4a,b). Furthermore, thickening of interventricular septum was noted in the AAB + HD group since W12, suggesting that hypercaloric diet associated with AAB hastens the occurrence of structural left ventricular modifications (left ventricular hypertrophy). This finding is supported by LV mass echocardiographic measurement at W36, when a significant (*p* < 0.05) increase was noted for the AAB + HD group compared to all the other three groups (Figure 4e).

Moreover, at the end of the study (W36), abdominal aortic stenosis lead to a significant (*p* < 0.05) increase in both LV end-diastolic (EDLV) and end-systolic (ESLV) volumes, compared to the Control group, regardless of the type of diet it was associated with (Figure 4c,d).

The chronic-discontinuous treatment with ALA (AAB + HD + ALA group) was able to prevent both PW and IVS thickening at W36 (Figure 4a,b); the effect on interventricular septum was more obvious and could be observed earlier, starting from W12. Although at 36 weeks, a trend (*p* > 0.05) of IVS thickening was noted in the AAB + HD + ALA group compared to Control, the IVS thickness in the AAB + HD + ALA group was significantly (*p* < 0.05) lower than the value obtained in the AAB + HD group. Moreover, ALA treatment attenuated the increase in end-systolic volume (W28) (Figure 4d), and preserved LV mass (W36) similar to the Control group (Figure 4e). However, in the case of end-systolic volume, the effect of ALA was no longer apparent at W36.

Aortic constriction, as measured at W36, was approximately 37% without significant differences between the three ligatured groups.

**Figure 5:**
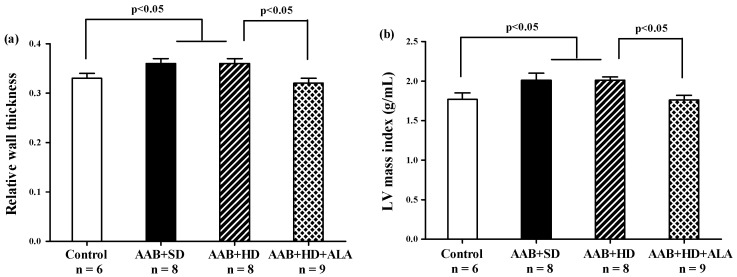
Hypertrophy echocardiographic markers at 36 weeks of study: (**a**) Relative wall thickness, (**b**) Left ventricular mass index (g/mL) (one-factor ANOVA).

Relative wall thickness (RWT) and LV mass index, two markers of cardiac concentric hypertrophy were significantly increased in the two ligatured groups (AAB + SD and AAB + HD), regardless of the used diet (Figure 5a,b). Additionally, the ALA-treated group presented with significantly lower RWT and LV mass index, which may suggest the delay of the development of cardiac concentric hypertrophy in this group (Figure 5a,b).

**Figure 6:**
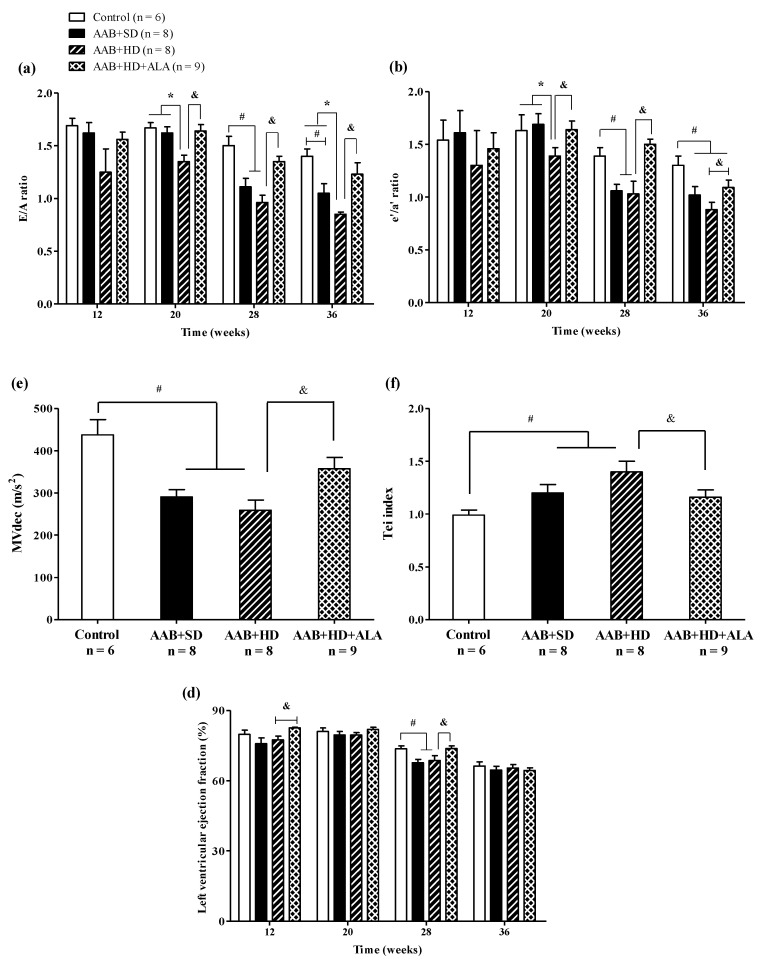
Evolution of functional echocardiographic parameters at 12-, 20-, 28- and 36-weeks of the study: (**a**) Early-to-late LV filling ratio (E/A); (**b**) Early-to-late LV diastolic relaxation ratio (e′/a′ratio); (**c**) Mitral valve flow deceleration (MVdec) evaluated at W36; (**d**) Tei index evaluated at W36 (**e**) Left ventricular ejection fraction (EF %). ^#^ *p* < 0.05: vs. Control, * *p* < 0.05: vs. AAB + SD and Control; ^&^ *p* < 0.05: vs. AAB + HD (one-factor ANOVA).

Regarding cardiac function, in the ligatured rats fed with hypercaloric diet (AAB + HD group) we noted a significant (*p* < 0.05) early decrease (W20) in early-to-late filling ratio (E/A) and early-to-late LV diastolic relaxation ratio (eʹ/aʹ); while for ligatured rats fed with standard diet (AAB + SD group), this decline occurred later, at W28 (Figure 6a,b). Furthermore, in both ligatured groups, LV filling inflow profile was significantly modified as seen also by mitral valve flow deceleration (MVdec) reduction (Figure 6c). Additionally, the Tei index, or myocardial performance index, a marker used to assess global heart dysfunction and prognostic in HFpEF patients, was found to be significantly (*p* < 0.05) increased in AAB + SD and AAB + HD groups (Figure 6d). However, the values of filling pressure, expressed as E/e′, were lower than 12 and were not significantly different between the four groups (data not shown).

Concerning systolic function, at W28 left ventricular ejection fraction (LVEF %) was found to be reduced with ~12% in AAB + SD and AAB + HD groups. Moreover, throughout the experiment, LVEF was > 60% in all groups and at the end of the experiment (W36), no difference between groups was observed concerning LVEF, which was found to be ~65% in all 4 groups (Figure 6e).

The chronic-discontinuous treatment with ALA (AAB + HD + ALA group) attenuated diastolic function decline by preventing the decrease in diastolic function parameters (E/A and eʹ/aʹ ratios since W20 and until the end of the experiment, and MVdec at W36) and the increase in Tei index (W36). Regarding LVEF, ALA treatment determined an increase in this parameter at W12 and W28 in comparison to the AAB + HD group. However, only a trend for increase (*p* = 0.07) was observed at W28, while at W36 the obtained values were similar for all four groups (Figure 6).

### 3.4. Blood/Plasma Measurements

#### 3.4.1. Biochemical Investigations

**Figure 7:**
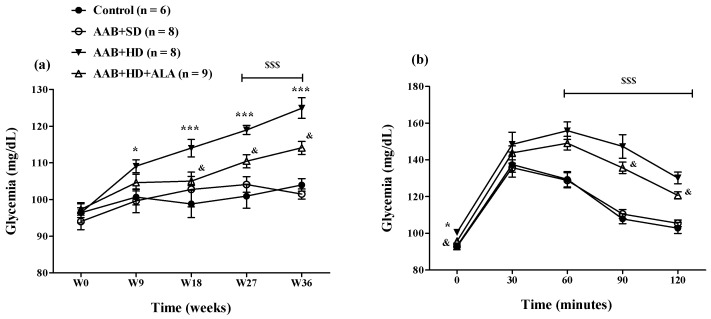
(**a**) Evolution of glycemia over a period of 36 weeks and (**b**) Evaluation of glycemia by the oral glucose tolerance test (OGTT) in W34 of the study. * *p* < 0.05, *** *p* < 0.001: AAB + HD vs. AAB + SD and Control groups; ^$$$^ *p* < 0.001: AAB + HD and AAB + HD + ALA vs. AAB + SD and Control groups; ^&^ *p* < 0.05: AAB + HD + ALA vs. AAB + HD (two-factor repeated measures ANOVA).

The hypercaloric diet intake (AAB + HD group) increased glycemia since W9 until the end of the study (Figure 7a). To better highlight this metabolic effect of hypercaloric diet, an oral glucose tolerance test (OGTT) was performed at W34 of the study (Figure 7b). During the OGTT, at 60, 90 and 120 min after oral glucose administration, glycemia values remained significantly higher (*p* < 0.001) in both groups fed with HD than the values recorded in the groups fed with standard diet (Figure 7b). The chronic discontinuous treatment with ALA alleviated the effect of HD on glycemia since W18, after the 4th sequence of treatment (Figure 7a). Moreover, during the OGTT test, at 90 and 120 min, in rats treated with ALA (AAB + HD + ALA group), glycemia values were inferior to those obtained in AAB + HD group, indirectly suggesting an increase in insulin efficacy (Figure 7b).

Moreover, at the end of the study, rats fed with hypercaloric diet (AAB + HD group) presented a significant (*p* < 0.01) hypertriglyceridemia; serum triglycerides were ~1.6 fold higher than in the groups fed with standard diet and HDL-cholesterol levels were decreased. In addition, the serum uric acid concentration was significantly (*p* < 0.01) decreased in ligatured rats (AAB + SD and AAB + HD groups) compared to the Control group. Additionally, no differences were observed between groups regarding serum transaminase activities.

In the long term, the chronic-discontinuous treatment with ALA prevented plasma lipoprotein profile alteration, especially influencing triglyceride and HDL-cholesterol levels, and also preventing the consumption of circulating uric acid (Table 1).

#### 3.4.2. Adiposity Markers

**Figure 8:**
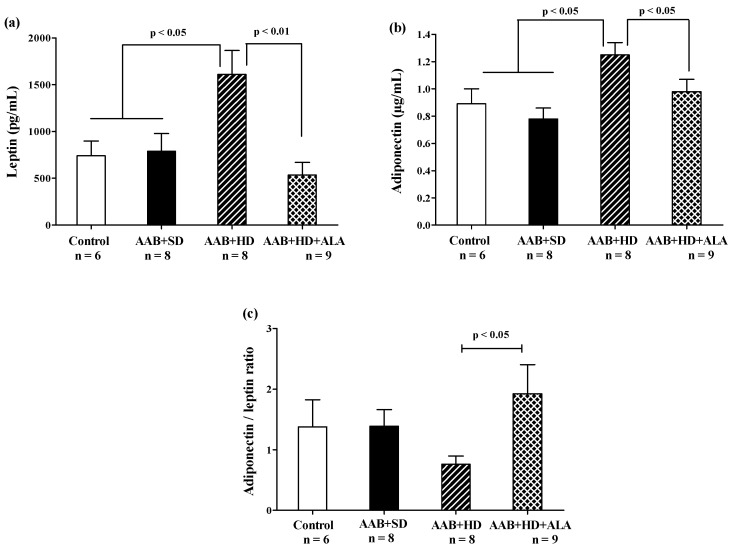
Adiposity markers: (**a**) Leptin, (**b**) Adiponectin and (**c**) Adiponectin/leptin ratio (one-factor ANOVA).

In the absence of treatment, hypercaloric diet (AAB + HD group) induced an increase in abdominal adipose tissue (Table 2) and plasma leptin and adiponectin (Figure 8a,b) concentrations compared to groups fed with standard diet (AAB + SD and Control). After 36 weeks, the chronic-discontinuous treatment with ALA was able to significantly reduce both abdominal adipose tissue (Table 2) and circulating leptin and adiponectin levels (Figure 8a,b), while significantly increasing adiponectin/leptin ratio (Figure 8c).

#### 3.4.3. Inflammatory Markers

**Figure 9:**
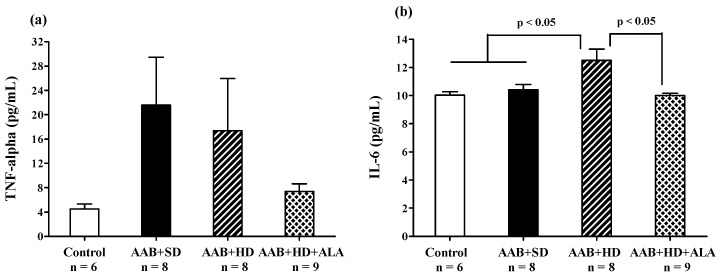
Plasma inflammatory markers: (**a**) TNF-α and (**b**) IL-6 (one-factor ANOVA).

Abdominal aortic banding (AAB + SD and AAB + HD groups) was associated with a tendency of increase in plasma TNF-α, regardless of the type of diet used (Figure 9a). Moreover, the plasma levels of IL-6 were significantly higher in the AAB + HD group compared to both groups fed with standard diet (AAB + SD and Control, Figure 9b). Other plasma inflammatory markers such as C-reactive protein (Table 1) did not register any differences in the four groups of animals studied. The chronic-discontinuous treatment with ALA was more effective (*p* < 0.05) in reducing the circulating levels of IL-6 (Figure 9b) and showed only a trend of reduction in TNF-α levels (Figure 9a).

#### 3.4.4. Brain Natriuretic Peptide

**Figure 10:**
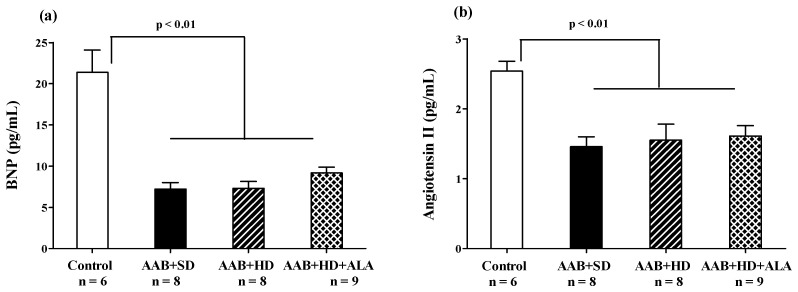
(**a**) Plasma Brain Natriuretic Peptide and (**b**) Plasma Angiotensin II levels (one-factor ANOVA).

At the end of the study (W36), in the three groups with abdominal aortic stenosis (AAB + SD, AAB + HD and AAB + HD + ALA), plasma levels of Brain Natriuretic Peptide (BNP) and Angiotensin II were significantly lower than the values obtained in the Control group (Figure 10a,b). The chronic-discontinuous treatment with ALA did not influence plasma BNP concentration (Figure 10a), nor that of Angiotensin II (Figure 10b).

#### 3.4.5. Oxidative Stress Markers

##### Plasma Lipid Peroxidation

**Figure 11:**
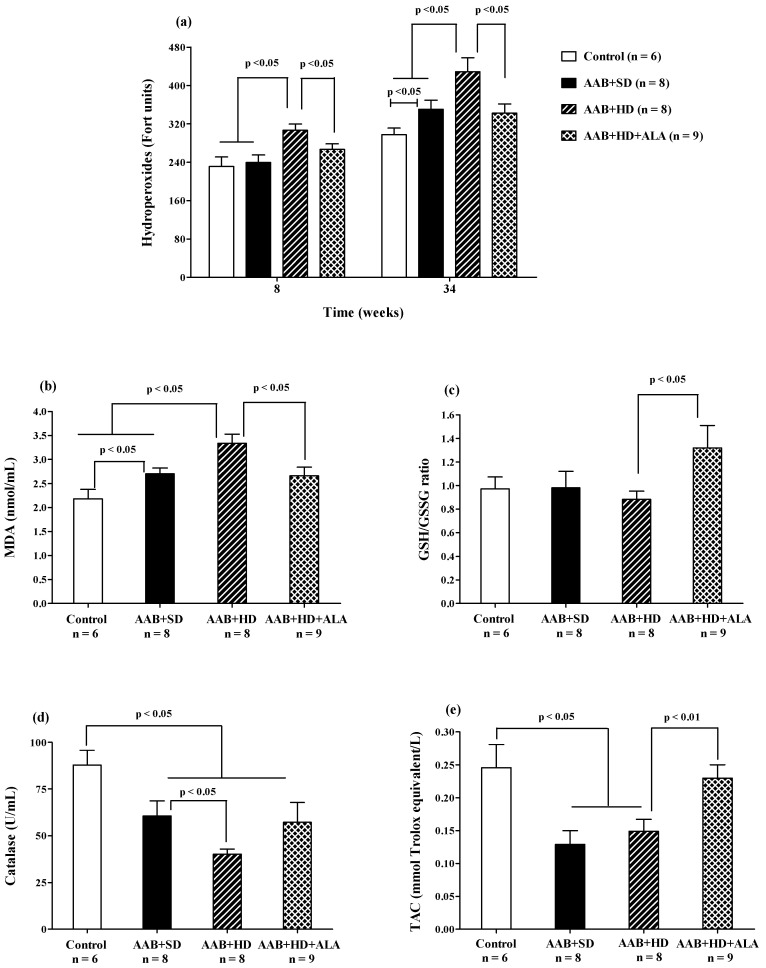
Oxidative stress markers: (**a**) Plasma Hydroperoxides (“FORT” test), (**b**) Plasma Malondialdehyde (MDA), (**c**) GSH/GSSG ratio, (**d**) Catalase and (**e**) Total antioxidant capacity (TAC) (one-factor ANOVA).

Plasma hydroperoxide (FORT test) levels increased after 8 weeks of hypercaloric diet (AAB + HD) compared to both groups fed with standard diet (AAB + SD and Control). Furthermore, at W34 of the study, in both ligatured groups (AAB + SD and AAB + HD) a significant increase in plasma hydroperoxide levels compared to the Control group was noted, but with significantly higher values in ligatured rats fed with hypercaloric diet (Figure 11a). Moreover, the same differences between groups were recorded for MDA, assessed at the end of the study (Figure 11b). ALA was able to prevent lipid peroxidation by decreasing both plasma hydroperoxides (Figure 11a), starting with W8 of the study, and MDA levels (W36, Figure 11b).

##### Circulating Antioxidants Levels

The aortic stenosis was associated with a decrease in plasma catalase activity (Figure 11d) and in total antioxidant capacity (TAC, Figure 11e) regardless of the type of diet, without influencing significantly the GSH/GSSG ratio (Figure 11c). Furthermore, the lowest values for catalase activity were recorded in AAB rats fed with hypercaloric diet (Figure 11d). The chronic-discontinuous treatment with ALA increased GSH/GSSG ratio (Figure 11c) and TAC (Figure 11e), without showing any effect on catalase activity (Figure 11d).

##### 3.4.6. Plasma NOx Levels

**Figure 12:**
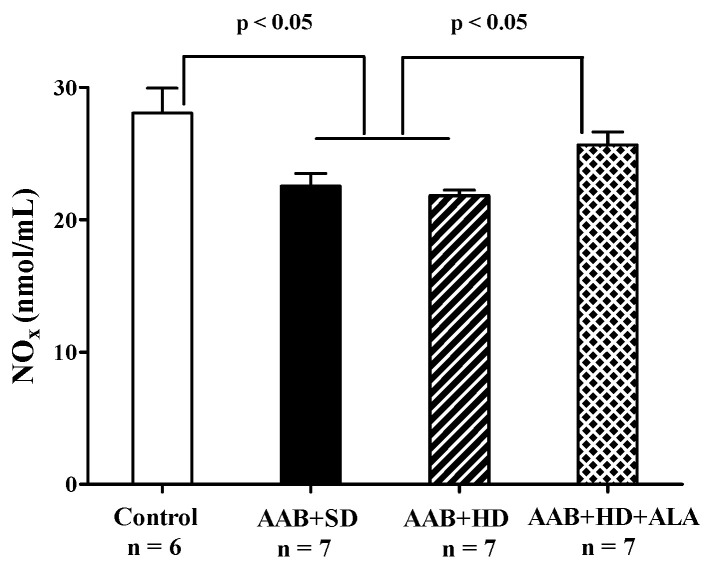
Plasma Nitrite and Nitrate levels (one-factor ANOVA).

Total plasma NO_x_ levels, biomarkers of NO bioavailability [41] were significantly decreased in both groups of ligatured rats regardless of the type of diet used. ALA discontinuous treatment prevented the decrease in this parameter, the measured levels being closer to those of the Control group (*p* > 0.05) (Figure 12).

### 3.5. Histological Analysis

**Figure 13:**
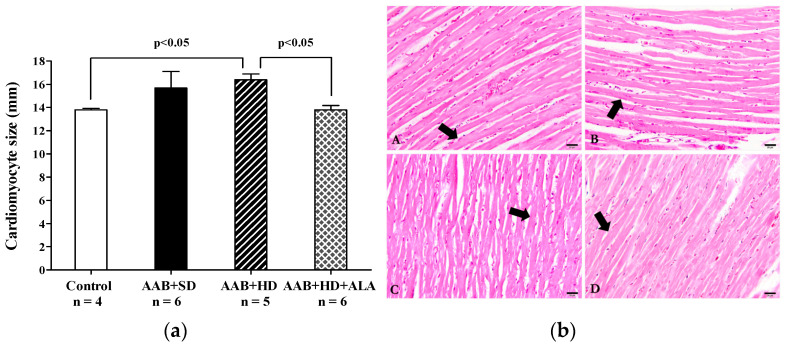
(**a**) Cardiac fibers diameters (one-factor ANOVA) and (**b**) Histological features of cardiac fibers in the experimental group. Myocardium fibers (arrow) from: A: AAB + SD group. B: AAB + HD group. C: AAB + HD + ALA group. D: Control group.

Concerning the myocardium, the main histological feature observed was the hypertrophy of the myocardial fibers in AAB + HD group compared to the Control group. Moreover, the chronic-discontinuous treatment with ALA was able to prevent the cardiac fibers hypertrophy (Figure 13a,b).

Regarding heart weight and heart weight/body weight (BW) ratio, no significant differences between the four groups of rats were recorded, regardless of AAB presence or diet type. However, the hypercaloric diet (AAB + HD group) induced a significant increase in abdominal adipose tissue and abdominal adipose tissue/BW ratio compared to standard food (AAB + SD), both parameters being significantly decreased when ALA treatment was associated with HD (Table 2).

## 4. Discussion

In our experimental conditions, the diet-induced obesity generated metabolic disorders, and in association with abdominal aortic stenosis it hastened the onset of heart failure, cardiac hypertrophy and diastolic dysfunction, all these disturbances being accompanied by a pro-oxidant and pro-inflammatory plasmatic status. Besides its antioxidant effects, the chronic discontinuous treatment with ALA prevented body weight gain and attenuated the metabolic and cardiac perturbations, confirming its protective effects on the cardiovascular system.

A high-fat diet (fat percentage >10%) or a high-fat high-carbohydrate diet (Western diet) are frequently used in animal experimental studies to reproduce body weight gain or obesity-associated cardiometabolic disorders (severe stages of metabolic syndrome or type 2 diabetes mellitus) [42,43,44,45]. In our study, the highest body weight was recorded in the rat groups fed with hypercaloric diet, which were overweight or even obese, although quantitatively they consumed less food than those fed with control diet, without any differences between the four groups regarding total caloric intake. Thus, it seems weight gain is highly influenced by nutritional quality of diet and macronutrient balance (balance between macronutrient intake and their oxidation) [46] than the calories intake per se [47,48]. SFA present in lard, the most effective and accessible type of fat for rat food [42,43] are oxidized less, providing lower diet-induced thermogenesis compared to the unsaturated ones, thus their storage being favored, with obvious consequences on body weight [42,45,47]. Because obesity is difficult to assess in animals, abdominal fat accumulation can confirm the obesity status [42], which was also reported in our study. Additionally, the presence of fructose in the diet inhibits fatty acids oxidation and amplifies hypertriglyceridemia, peripheral insulin resistance and visceral adiposity [17,42,49,50]. Although a hypercaloric diet generates carbohydrate and lipid metabolism disruptions and systolic blood pressure increase, Vileigas et al. show that cardiac function impairment appears only in the western diet with higher fat predominance (high fat western diet) [47], emphasizing the effect of diet composition on cardiac risk.

Besides its role in energy storage, adipose tissue is an important endocrine organ, which influences a variety of physiological and pathological processes via the multiple secreted adipokines [51,52]. Plasma leptin is directly proportional to the amount of adipose tissue; it is involved in regulating body energy balance and also presents pro-inflammatory and pro-oxidant properties, and promotes cardiac fibrosis and hypertrophy [51]. In turn, adiponectin, frequently found in low concentrations in obese persons, exhibits anti-inflammatory and cardioprotective effects and improves insulin sensitivity [53]. Despite all these beneficial effects, recent studies paradoxically found high circulating adiponectin levels associated with an increased cardiovascular mortality risk (the adiponectin paradox) [53,54]. In our study, plasmatic leptin was higher in obese rats (AAB + HD group), accompanied by an unexpected increase in adiponectin and a declining trend for adiponectin/leptin ratio. There are also other studies reporting high levels of adiponectin in overweight or obesity conditions [55,56,57,58], so far without offering a clear mechanism responsible for these findings. It is possible that during the early stages of weight gain, there is a leptin-dependent up-regulation of adiponectin expression, a process maintained until the reach of a “threshold” of leptin action, associated with impairment of leptin signaling. From this moment on, the leptin-adiponectin ratio is reversed with a significant enhancement in leptin and decline in adiponectin levels. This situation is not always reached in the same timeframe for all individuals, depending on inter-individual variability and experimental conditions [59]. This could explain, at least partially, the contradictory results regarding adiponectin levels.

Our chronic discontinuous treatment with ALA prevented the rats’ weight gain although the food pellet consumption and energy intake were similar in the two groups fed with hypercaloric diet. In this case the anti-obesity effect of ALA could be a consequence of the increase in energy expenditure, especially as ALA stimulates both cellular glucose uptake [60,61,62] and ATP synthesis, being an important cofactor of mitochondrial pyruvate and ketoglutarate dehydrogenases [63,64]. Furthermore, several studies have noted that ALA activates skeletal muscle 5’ adenosine monophosphate–activated protein kinase (AMPK), with a subsequent enhancement of fatty acid β-oxidation [25,65] and increases mitochondrial uncoupling protein (UCP)-1 expression in adipose tissue, which has an important role in non-shivering thermogenesis [24]. Therefore, it could be assumed that by these two combined mechanisms, ALA increases whole-body energy expenditure [66], stimulating weight loss or preventing weight gain, depending on the initial body weight at the moment of treatment. The good control of obesity under ALA treatment could also be derived from low abdominal fat and an increase in adiponectin/leptin ratio. This ratio is inversely correlated with adipose tissue dysfunction, characterized by subclinical inflammatory state, insulin resistance and a high risk for metabolic syndrome [67,68]. Improving this parameter could be a valuable way to control obesity-associated cardiometabolic alterations [69].

HFpEF is difficult to diagnose in animals and so far, there is no ideal experimental model that reproduces this human heterogeneous cardiac pathology [70,71,72,73]. Moderate aortic banding, applied at an early age, produces a constriction that gradually advances as animals grow, first inducing an increase in blood pressure proximal to the region of stenosis, increased afterload, followed by cardiac hypertrophy development and ultimately ventricular function impairment [5,70]. For this reason, AAB experimental conditions are much more similar to the evolution of human heart failure induced by arterial hypertension [72]. Moreover, our experimental conditions associate AAB with another major cardiovascular risk factor, namely obesity, induced by hypercaloric diet, thus closely reproducing the context of obesity-related HFpEF [1,74]. More recently, Schiattarella et al. [75] reported a non-invasive way to obtain simultaneous metabolic and hypertensive stress that leads to earlier development of HFpEF features in rodents, by combining a high-fat diet with an inhibitor of endothelial nitric oxide synthase (eNOS). This model caused a more advanced state of diastolic dysfunction similar to a restrictive pattern (E/A > 2 and significantly increased in E/e′ ratio, at 15W), while our experimental conditions produced a mild form of diastolic dysfunction with normal filling pressure of the left ventricle (E/A < 1 but without significant increase in E/e′ ratio, at 36W) [76]. Therefore, the model of HFpEF that we used may be more appropriate for exploring the benefits of long-term preventive treatment with potential therapeutic agents, while the other one could prove optimal for testing more targeted therapies, with faster-obtained preliminary results.

In our present study, a hypercaloric diet together with AAB induced significant echocardiographic structural and functional abnormalities. Thus, the interventricular septum and posterior wall thickening associated with LV volumes and LV mass increases were clearly observed at 36 weeks. However, as the interventricular septum is highly susceptible to pressure-overload [77], its thickening was recorded earlier (12 weeks), followed ultimately by the posterior wall hypertrophy (36 weeks). Moreover, a higher LV mass index and relative wall thickness in the ligatured groups, associated with an increase in transverse cardiomyocytes diameter (short-axis diameter), observed by histological examination, confirmed the presence of concentric LV hypertrophy.

LV hypertrophy is frequently associated with diastolic dysfunction in experimental and clinical studies, both representing the hallmarks of HFpEF [2,8]. Regarding diastolic dysfunction, our data revealed LV passive filling (E/A ratio < 1) and active LV relaxation (e′/a′ ratio < 1) impairment, accompanied by a significant decrease in mitral valve flow deceleration (MVdec). Furthermore, decreased E/A and e′/a′ ratios were significantly accelerated by the association of HD to AAB (20 weeks for AAB + HD group versus 28 weeks for AAB + SD group). Another important finding was the increase in the myocardial performance index, or Tei index (at W36, regardless of the diet used), notably used to assess diastolic dysfunction and for prognostic information in patients with HFpEF [78]. Although these parameters are relevant to diastolic dysfunction, its diagnosis remains challenging, especially in experimental models [79]. Moreover, in our study moderate changes in LVEF were observed, but without systolic function alterations (EF > 50%). In murine models, the period of transition to systolic dysfunction and HFrEF can be variable and represents a valuable feature which is not yet very well documented in humans [72,73]. In some experimental studies, HFpEF preceded the later onset of HFrEF [72], while other studies reported a rapid decrease in ejection fraction (EF < 40%) after aortic stenosis (AAB) with a significant loss in animals’ body weight in some situations [28,32,80]. These differences in cardiovascular phenotype obtained after abdominal aortic stenosis [28,32,71,72,80] could be mainly attributed to the specific genetic background of animals or to the rat strains (Wistar rats versus Sprague-Dawley rats) and to the types and compositions of diets used.

Even if at the end of the study all AAB rats were characterized by cardiac hypertrophy and diastolic dysfunction with preserved EF, circulating BNP levels were found to be below the values recorded for the control group. However, in patients with higher body weight mass index (BMI) or diabetes mellitus, disproportionately low levels of natriuretic peptides (NPs) have been reported. These values were lower than traditional diagnostic cut-off reference values for HF (BNP > 35 pg/mL) [1] or than in the normoponderal persons [74,81,82]. Thus, considering this accumulation of data and the fact that obese people with signs and symptoms of HF but with low levels of BNP have been generally excluded from many trials [7,81], a new obesity-related HFpEF phenotype has been proposed [6,74]. These low levels of NPs could be explained by the limited cardiomyocyte stretch, specific to HFpEF with diastolic dysfunction [83] overlapped with the excess adipose tissue, responsible for both overexpression of natriuretic peptide clearance receptors (NPRC) and increased neprilysin secretion [11,82]. Neprilysin is also secreted by proximal renal tubular epithelial cells, in response to sympathetic nerve stimulation [11,84], that can occur in the presence of abdominal aortic stenosis. In our experiment, increased neprilysin secretion would be the key element that could explain the decreased BNP and AgII levels in all AAB groups. However, these hypotheses remain open issues. Further studies showing BNP, NT pro-BNP and neprilysin concentrations at different moments over the period of the study are needed.

During our study, ALA chronic discontinuous treatment showed beneficial effects on two of the main features of HFpEF, namely cardiac hypertrophy and diastolic dysfunction, without significant effects on circulating BNP. Due to SBP attenuation and weight gain prevention, ALA could decrease cardiac afterload and inhibit cardiac hypertrophy highlighted both by echocardiography parameters (relative wall thickness and LV mass index) and by histopathological analysis (cardiomyocytes’ dimensions). Moreover, ALA treatment delayed LV relaxation and passive filling impairments and overall inhibited the increase in cardiac performance index (Tei index), thus delaying diastolic dysfunction installation. However, the effects of ALA on certain cardiac parameters (end-systolic LV volume, LVEF) are less consistent, at different time points. As no additional intervention (dietary restriction, and/or modification to ALA dose or rhythm of administration) has been done during the experiment, it is possible that the protective abilities of ALA were overwhelmed by the accumulation of pressure overload. This could also help explain the trend of IVS thickening that can be observed in the ALA-treated group versus Control at W36, especially considering that IVS is highly susceptible to pressure-overload hypertrophy [77]. Additionally, differences in EF values can occur due to animals’ reactivity to anesthesia, conferring a high variability to this parameter. This, together with the limited number of animals/group could explain why statistical significance was not attained for LVEF at W20 and W36. Nevertheless, when EF values are higher than 50%, differences in this parameter are not considered to have clinical significance, for both diagnosis and treatment efficacy [1].

Obesity-related HFpEF obtained in our experimental conditions was associated with a subclinical pro-inflammatory status highlighted by significantly increased IL-6 and leptin plasma levels, a trend for increased TNF-alpha and decreased adiponectin/leptin ratio, but without CRP modifications. The main mechanism by which IL-6 and TNF-alpha influence diastolic function could be impairment of reticular Ca^2+^ reuptake due to SERCA2 (sarcoplasmic reticulum Ca^2+^-ATPase) downregulation in cardiomyocytes, thus increasing ventricular stiffness [85]. Additionally, TNF-alpha stimulates the NF-κB pathway, leading to cardiomyocyte concentric hypertrophy [18,86,87,88]. The higher expected levels of TNF-alpha and possibly the general pro-inflammatory state observed in ligatured rats fed a hypercaloric diet could, at least partially be attenuated by the high levels of adiponectin that were found in this experimental group. The observed subclinical inflammation was significantly attenuated by ALA discontinuous treatment, complementing previously reported data that revealed the anti-inflammatory properties of this molecule [22]. ALA can determine anti-inflammatory effects by both direct mechanisms, via down-regulation of the NF-kB pathway, and indirect mechanisms involving improvement of redox status with decreases in pro-inflammatory cytokines [22,62,89,90].

In addition to the pro-inflammatory state observed in HFpEF, the occurrence of oxidative stress (OS) has also been highlighted as a key element in the pathophysiology of this condition [91]. In HF, OS was shown to cause direct cellular damage but also to affect excitation–contraction coupling and to promote myocardial remodeling [92]. Furthermore, decreased GSH levels were shown to be correlated with increased TNF-alpha [93], confirming that OS and inflammation co-exist and amplify each other in HFpEF [91]. Our data are in accordance with these findings, since rats suffering from HD-induced obesity and HFpEF, showed an impaired antioxidant status (downward trend for the GSH/GSSG ratio with a significant decrease in total antioxidant capacity, catalase activity and uric acid levels), and increased oxidative damage, measured as circulating MDA, and hydroperoxides as well as a pro-inflammatory status. Furthermore, in the case of hydroperoxides, at W8, before any signs of HFpEF were apparent, the hypercaloric diet by itself caused an increase in this biomarker. Subsequently, at W34, an additive effect could be observed for the hypercaloric diet and HFpEF, since increased levels of hydroperoxides were determined in the AAB + SD group, but the increase was significantly greater in the AAB + HD rats. A similar pattern was noted for MDA at the end of the study (W36).

It was postulated that decreased NO bioavailability in the endothelium is the main mechanism involved in HFpEF. This is caused by the uncoupling of eNOS and the increase in O_2_^•−^ synthesis with subsequent formation of highly reactive peroxynitrite (ONOO^−^) [94]. Our results are in agreement with this hypothesis, since NO bioavailability, measured as total plasmatic nitrites and nitrates, was decreased in both ligatured groups. One of the consequences of reduced NO bioavailability is the decrease in cGMP levels. The alternative pathway for cGMP formation is BNP-dependent [94]. Therefore, in our model of obesity-related HFpEF, the association of OS and lower BNP concentrations could have had an additive effect on cGMP concentrations. Independently of NO bioavailability this signaling pathway can be dysregulated by sGC oxidation, which renders sGC insensitive to NO, and by cardiac fibrosis [95].

The majority of redox perturbations that we observed in the HD + AAB experimental group were attenuated by a discontinuous treatment with ALA, which improved antioxidant status and decreased circulating levels of MDA and hydroperoxides. For the latter, this effect was already observed after two rounds of treatment. This is in accordance with previously published data, showing that ALA can prevent lipid peroxidation and regenerate endogenous antioxidants [89,96]. Besides the direct reduction of GSSG to GSH, ALA can also increase GSH synthesis via the activation of the nuclear factor erythroid 2-related factor 2 (Nrf2) pathway and/or the increase in cysteine levels (increased uptake, reduction in cystine) [22,62]. This could explain the increased GSH/GSSG ratio and total antioxidant capacity (mainly non-enzymatic antioxidants [97]) that we found in ALA-treated animals. Furthermore, it has been proven that ALA can regulate the expression of O_2_^•−^ producing enzymes, such as NADPH oxidase and increase the expression of superoxide dismutase and thioredoxin [96]. Lower formation of O_2_^•−^ and increased antioxidant defenses induced by ALA would justify the reported decrease in oxidative damage markers. The antioxidant activity of ALA together with its effect on circulating NO levels could explain the observed cardioprotective effects.

The benefits of the long-term discontinuous treatment with ALA, highlighted in this study, may be partially related to the formation of dihydrolipoic acid (DHLA), the intracellular active metabolite, generating the ALA/DHLA antioxidant couple, which can be recycled over longer periods of time, via NADH [90,98] and to the regulation of cellular redox status and signaling pathways/transcription factors by both of them [63]. Moreover, the capacity of some tissues (i.e., heart, kidney, liver, skeletal muscle) to store ALA as lypolysine [22,62] may prolong its effects during the break between treatment sequences. The use of a discontinuous treatment could improve patient adherence, especially in the case of long-term therapy. However, further studies are needed before translating experimental results to clinical settings.

The main limitations of our study could be the lack of data regarding plasmatic neprilysin or NT-proBNP (N-terminal prohormone of brain natriuretic peptide) concentrations and histological analysis of cardiac fibrosis. Measuring plasma neprilysin or NT-proBNP could help explain the decreased circulating BNP and Angiotensin II observed in our study. The histological analysis could help evaluate the severity of HFpEF and better understand the observed cardioprotective effects of ALA. Furthermore, studies showing measurements of oxidative stress and inflammation biomarkers in cardiac tissue could bring new insight and support the use of ALA in cardiometabolic disorders.

## 5. Conclusions

In our experimental conditions, near-normal ejection fraction together with diastolic dysfunction and cardiac hypertrophy confirm the development of HFpEF induced by abdominal aortic stenosis. Additionally, the associated diet-induced obesity hastens the onset of these perturbations and increases their severity. The obtained experimental model could prove more appropriate than the ones previously reported for studying the particular phenotype of obesity-related HFpEF. ALA discontinuous treatment prevented weight gain and attenuated glucose and lipid metabolism disturbances. Furthermore, it prevented the development of cardiac hypertrophy and it both attenuated and delayed the onset of diastolic dysfunction. The observed cardioprotective effects of ALA could be justified by its known antioxidant effects, as well as intrinsic anti-obesity and anti-inflammatory properties, that were also highlighted in our experiment. The chronic discontinuous treatment regimen could be easier to translate to clinical settings, given the better long-term patient adherence to treatment. Therefore, the results obtained in our study show the promising potential of ALA treatment in cardiometabolic disorders and further confirm the pleiotropic effects of this antioxidant molecule in HFpEF.

## Figures and Tables

**Table 1:** Serum levels of plasma lipoproteins, uric acid, C-reactive protein and transaminase activities, assessed after 36 weeks of study.

Parameters	Control (*n* = 6)	AAB + SD (*n* = 8)	AAB + HD (*n* = 8)	AAB + HD + ALA (*n* = 9)
total Cholesterol (mg/dL)	72.40 ± 4.31	69.13 ± 5.13	81.80 ± 6.68	71.86 ± 3.13
LDL-Cholesterol (mg/dL)	11.58 ± 1.06	12.83 ± 0.77	15.60 ± 1.68	11.63 ± 1.16
HDL-Cholesterol (mg/dL)	68.57 ± 4.29	64.80 ± 4.08	54.33 ± 4.22 ^#^	69.50 ± 2.78 ^&^
Triglycerides (mg/dL)	95.36 ± 11.80	90.00 ± 9.07	152.00 ± 22.27 **	87.00 ± 10.14 ^&&^
ASAT (U/L)	142.63 ± 5.41	141.40 ± 7.01	139.00 ± 9.06	137.25 ± 17.97
ALAT (U/L)	39.13 ± 2.69	38.00 ± 3.89	42.33 ± 6.73	39.88 ± 3.84
Uric acid (mg/dL)	3.16 ± 0.25	2.48 ± 0.2 ^#^	2.03 ± 0.16 ^##^	2.61 ± 0.23 ^&^
C-reactive protein (mg/dL)	0.46 ± 0.03	0.47 ± 0.01	0.49 ± 0.03	0.44 ± 0.02
Creatinine (mg/dL)	0.39 ± 0.01	0.37 ± 0.02	0.35 ± 0.01 ^#^	0.36 ± 0.05

^#^*p* < 0.05, ^##^ *p* < 0.01: vs. Control; ** *p* < 0.01: vs. AAB + SD and Control; ^&^ *p* < 0.05, ^&&^ *p* < 0.01: vs. AAB + HD (one-factor ANOVA).

**Table 2:** Organ weight in the evaluated rat groups.

Organ Weight	Control (*n* = 6)	AAB + SD (*n* = 8)	AAB + HD (*n* = 8)	AAB + HD + ALA (*n* = 9)
Heart (g)	1.29 ± 0.05	1.38 ± 0.04	1.42 ± 0.06	1.33 ± 0.07
Heart/BW Ratio (g/kg)	0.22 ± 0.01	0.23 ± 0.01	0.20 ± 0.01 *	0.22 ± 0.01 ^&^
Abdominal adipose tissue (g)	23.65 ± 3.26	19.26 ± 3.49	48.46 ± 4.52 **	25.56 ± 4.60 ^&&^
Abdominal adipose tissue/BW ratio (g/kg)	4.02 ± 0.56	3.16 ± 0.44	6.86 ± 0.61 *	4.15 ± 0.60 ^&&^

* *p* < 0.05, ** *p* < 0.01: vs. AAB + SD and Control; ^&^ *p* < 0.05, ^&&^ *p* < 0.01: AAB + HD + ALA vs. AAB + HD (one-factor ANOVA)**.**

## References

[1] Ponikowski P., Voors A.A., Anker S.D., Bueno H., Cleland J.G.F., Coats A.J.S., Falk V., González-Juanatey J.R., Harjola V.P., Jankowska E.A. (2016). 2016 ESC Guidelines for the diagnosis and treatment of acute and chronic heart failure. Eur. Heart J..

[2] van Heerebeek L., Paulus W.J. (2016). Understanding heart failure with preserved ejection fraction: Where are we today?. Neth. Heart J..

[3] Salah E.M., Bastacky S.I., Jackson E.K., Tofovic S.P. (2018). Captopril Attenuates Cardiovascular and Renal Disease in a Rat Model of Heart Failure with Preserved Ejection Fraction. J. Cardiovasc. Pharmacol..

[4] Tadic M., Cuspidi C. (2019). Obesity and heart failure with preserved ejection fraction: A paradox or something else?. Heart Fail. Rev..

[5] Yoon S., Eom G.H. (2019). Heart failure with preserved ejection fraction: Present status and future directions. Exp. Mol. Med..

[6] Komajda M., Lam C.S.P. (2014). Heart failure with preserved ejection fraction: A clinical dilemma. Eur. Heart J..

[7] Meijers W.C., Hoekstra T., Jaarsma T., Van Veldhuisen D.J., de Boer R.A. (2016). Patients with heart failure with preserved ejection fraction and low levels of natriuretic peptides. Neth. Heart J..

[8] Borlaug B.A., Paulus W.J. (2011). Heart failure with preserved ejection fraction: Pathophysiology, diagnosis, and treatment. Eur. Heart J..

[9] Obokata M., Reddy Y.N.V., Pislaru S.V., Melenovsky V., Borlaug B.A. (2017). Evidence Supporting the Existence of a Distinct Obese Phenotype of Heart Failure with Preserved Ejection Fraction. Circulation.

[10] Savji N., Meijers W.C., Bartz T.M., Bhambhani V., Cushman M., Nayor M., Kizer J.R., Sarma A., Blaha M.J., Gansevoort R.T. (2018). The Association of Obesity and Cardiometabolic Traits With Incident HFpEF and HFrEF. JACC Heart Fail..

[11] Packer M. (2018). Leptin-Aldosterone-Neprilysin Axis: Identification of Its Distinctive Role in the Pathogenesis of the Three Phenotypes of Heart Failure in People with Obesity. Circulation.

[12] Paulus W.J., Tschöpe C. (2013). A novel paradigm for heart failure with preserved ejection fraction: Comorbidities drive myocardial dysfunction and remodeling through coronary microvascular endothelial inflammation. J. Am. Coll. Cardiol..

[13] Riehle C., Bauersachs J. (2019). Key inflammatory mechanisms underlying heart failure. Herz.

[14] Van Empel V., Brunner-La Rocca H.P. (2015). Inflammation in HFpEF: Key or circumstantial?. Int. J. Cardiol..

[15] Lucas S., Verwaerde C., Wolowczuk I. (2009). Is the adipose tissue the key road to inflammation?. Immunol. Immunogenet. Insights.

[16] Engin A., Engin A.B., Engin A. (2017). The Pathogenesis of Obesity-Associated Adipose Tissue Inflammation. Obesity and Lipotoxicity.

[17] Ghibu S., Craciun C.E., Rusu R., Morgovan C., Mogosan C., Rochette L., Gal A.F., Dronca M. (2019). Impact of Alpha-Lipoic Acid Chronic Discontinuous Treatment in Cardiometabolic Disorders and Oxidative Stress Induced by Fructose Intake in Rats. Antioxidants.

[18] Ayoub K.F., Pothineni N.V.K., Rutland J., Ding Z., Mehta J.L. (2017). Immunity, Inflammation, and Oxidative Stress in Heart Failure: Emerging Molecular Targets. Cardiovasc. Drugs Ther..

[19] Gevaert A.B., Boen J.R.A., Segers V.F., Van Craenenbroeck E.M. (2019). Heart failure with preserved ejection fraction: A review of cardiac and noncardiac pathophysiology. Front. Physiol..

[20] Hopps E., Noto D., Caimi G., Averna M.R. (2010). A novel component of the metabolic syndrome: The oxidative stress. Nutr. Metab. Cardiovasc. Dis..

[21] Roberts C.K., Sindhu K.K. (2009). Oxidative stress and metabolic syndrome. Life Sci..

[22] Rochette L., Ghibu S., Muresan A., Vergely C. (2015). Alpha-lipoic acid: Molecular mechanisms and therapeutic potential in diabetes. Can. J. Physiol. Pharmacol..

[23] Cremer D.R., Rabeler R., Roberts A., Lynch B. (2006). Safety evaluation of alpha-lipoic acid (ALA). Regul. Toxicol. Pharmacol..

[24] El Midaoui A., Fantus I.G., Boughrous A.A., Couture R. (2019). Beneficial effects of alpha-lipoic acid on hypertension, visceral obesity, UCP-1 expression and oxidative stress in zucker diabetic fatty rats. Antioxidants.

[25] Gomes M.B., Negrato C.A. (2014). Alpha-lipoic acid as a pleiotropic compound with potential therapeutic use in diabetes and other chronic diseases. Diabetol. Metab. Syndr..

[26] Prieto-Hontoria P.L., Pérez-Matute P., Fernández-Galilea M., Martínez J.A., Moreno-Aliaga M.J. (2013). Effects of lipoic acid on AMPK and adiponectin in adipose tissue of low- and high-fat-fed rats. Eur. J. Nutr..

[27] Namazi N., Larijani B., Azadbakht L. (2018). Alpha-lipoic acid supplement in obesity treatment: A systematic review and meta-analysis of clinical trials. Clin. Nutr..

[28] Pop C., Berce C., Ghibu S., Scurtu I., Soritău O., Login C., Kiss B., Stefan M.G., Fizesan I., Silaghi H. (2020). Effects of Lycium barbarum L. Polysaccharides on inflammation and oxidative stress markers in a pressure overload-induced heart failure rat model. Molecules.

[29] Pop C., Berce C., Ghibu S., Pop A., Kiss B., Irimie A., Popa Ş., Cismaru G., Loghin F., Mogoşan C. (2016). Validation and characterization of a heart failure animal model. Farmacia.

[30] Lang R.M., Bierig M., Devereux R.B., Flachskampf F.A., Foster E., Pellikka P.A., Picard M.H., Roman M.J., Seward J., Shanewise J. (2006). Recommendations for chamber quantification. Eur. J. Echocardiogr..

[31] Reffelmann T., Hale S.L., Dow J.S., Kloner R.A. (2003). No-Reflow Phenomenon Persists Long-Term after Ischemia/Reperfusion in the Rat and Predicts Infarct Expansion. Circulation.

[32] Rouhana S., Farah C., Roy J., Finan A., Rodrigues de Araujo G., Bideaux P., Scheuermann V., Saliba Y., Reboul C., Cazorla O. (2019). Early calcium handling imbalance in pressure overload-induced heart failure with nearly normal left ventricular ejection fraction. Biochim. Biophys. Acta Mol. Basis Dis..

[33] Panchal S.K., Poudyal H., Iyer A., Nazer R., Alam A., Diwan V., Kauter K., Sernia C., Campbell F., Ward L. (2011). High-carbohydrate high-fat diet-induced metabolic syndrome and cardiovascular remodeling in rats. J. Cardiovasc. Pharmacol..

[34] Pavlatou M.G., Papastamataki M., Apostolakou F., Papassotiriou I., Tentolouris N. (2009). FORT and FORD: Two simple and rapid assays in the evaluation of oxidative stress in patients with type 2 diabetes mellitus. Metabolism.

[35] Ghibu S., Delemasure S., Richard C., Guilland J.-C., Martin L., Gambert S., Rochette L., Vergely C. (2012). General oxidative stress during doxorubicin-induced cardiotoxicity in rats: Absence of cardioprotection and low antioxidant efficiency of alpha-lipoic acid. Biochimie.

[36] Porfire A.S., Leucuţa S.E., Kiss B., Loghin F., Pârvu A.E. (2014). Investigation into the role of Cu/Zn-SOD delivery system on its antioxidant and antiinflammatory activity in rat model of peritonitis. Pharmacol. Rep..

[37] Aebi H. (1984). Catalase in Vitro. Methods Enzymol..

[38] Erel O. (2004). A novel automated direct measurement method for total antioxidant capacity using a new generation, more stable ABTS radical cation. Clin. Biochem..

[39] Miranda K.M., Espey M.G., Wink D.A. (2001). A Rapid, Simple Spectrophotometric Method for Simultaneous Detection of Nitrate and Nitrite. Nitric Oxide.

[40] Rusu M.E., Georgiu C., Pop A., Mocan A., Kiss B., Vostinaru O., Fizesan I., Stefan M.-G., Gheldiu A.-M., Mates L. (2020). Antioxidant Effects of Walnut (*Juglans regia* L.) Kernel and Walnut Septum Extract in a D-Galactose-Induced Aging Model and in Naturally Aged Rats. Antioxidants.

[41] Chirinos J.A., Akers S.R., Trieu L., Ischiropoulos H., Doulias P.T., Tariq A., Vassim I., Koppula M.R., Syed A.A., Soto-Calderon H. (2016). Heart failure, left ventricular remodeling, and circulating nitric oxide metabolites. J. Am. Heart Assoc..

[42] Rodríguez-Correa E., González-Pérez I., Clavel-Pérez P.I., Contreras-Vargas Y., Carvajal K. (2020). Biochemical and nutritional overview of diet-induced metabolic syndrome models in rats: What is the best choice?. Nutr. Diabetes.

[43] Buettner R., Parhofer K.G., Woenckhaus M., Wrede C.E., Kunz-Schughart L.A., Schölmerich J., Bollheimer L.C. (2006). Defining high-fat-diet rat models: Metabolic and molecular effects of different fat types. J. Mol. Endocrinol..

[44] Moreno-Fernández S., Garcés-Rimón M., Vera G., Astier J., Landrier J., Miguel M. (2018). High Fat/High Glucose Diet Induces Metabolic Syndrome in an Experimental Rat Model. Nutrients.

[45] Ghibu S., Ilie I., Mureșan A., Mogoșan C. (2015). Perspectives in the experimental study of the metabolic syndrome. Farmacia.

[46] Krishnan S., Cooper J.A. (2014). Effect of dietary fatty acid composition on substrate utilization and body weight maintenance in humans. Eur. J. Nutr..

[47] Vileigas D.F., de Carvalho Marciano C.L., Mota G.A.F., de Souza S.L.B., Sant’Ana P.G., Okoshi K., Padovani C.R., Cicogna A.C. (2019). Temporal Measures in Cardiac Structure and Function During the Development of Obesity Induced by Different Types of Western Diet in a Rat Model. Nutrients.

[48] Bortolin R.C., Vargas A.R., Gasparotto J., Chaves P.R., Schnorr C.E., Martinello K.B., Silveira A.K., Rabelo T.K., Gelain D.P., Moreira J.C.F. (2018). A new animal diet based on human Western diet is a robust diet-induced obesity model: Comparison to high-fat and cafeteria diets in term of metabolic and gut microbiota disruption. Int. J. Obes..

[49] Ghibu S., Decea N., Morgovan C., Mogosan C. (2013). An experimental model to induce metabolic syndrome in Rats. The fructose-enriched diet. Farmacia.

[50] Hannou S.A., Haslam D.E., McKeown N.M., Herman M.A. (2018). Fructose metabolism and metabolic disease. J. Clin. Investig..

[51] Landecho M.F., Tuero C., Valentí V., Bilbao I., de la Higuera M., Frühbeck G. (2019). Relevance of leptin and other adipokines in obesity-associated cardiovascular risk. Nutrients.

[52] Taube A., Schlich R., Sell H., Eckardt K., Eckel J. (2012). Inflammation and metabolic dysfunction: Links to cardiovascular diseases. Am. J. Physiol. Heart Circ. Physiol..

[53] Antoniades C., Antonopoulos A.S., Tousoulis D., Stefanadis C. (2009). Adiponectin: From obesity to cardiovascular disease: Etiology and Pathophysiology. Obes. Rev..

[54] Menzaghi C., Trischitta V. (2018). The adiponectin paradox for all-cause and cardiovascular mortality. Diabetes.

[55] Aslam M., Madhu S.V. (2017). Development of metabolic syndrome in high-sucrose diet fed rats is not associated with decrease in adiponectin levels. Endocrine.

[56] Kamari Y., Grossman E., Oron-Herman M., Peleg E., Shabtay Z., Shamiss A., Sharabi Y. (2007). Metabolic stress with a high carbohydrate diet increases adiponectin levels. Horm. Metab. Res..

[57] Cano P., Cardinali D.P., Ríos-Lugo M.J., Fernández-Mateos M.P., Reyes Toso C.F., Esquifino A.I. (2009). Effect of a high-fat diet on 24-hour pattern of circulating adipocytokines in rats. Obesity.

[58] Garcés-Rimón M., González C., Uranga J.A., López-Miranda V., López-Fandiño R., Miguel M. (2016). Pepsin egg white hydrolysate ameliorates obesity-related oxidative stress, inflammation and steatosis in Zucker fatty rats. PLoS ONE.

[59] Singh P., Sharma P., Sahakyan K.R., Davison D.E., Sert-Kuniyoshi F.H., Romero-Corral A., Swain J.M., Jensen M.D., Lopez-Jimenez F., Kara T. (2016). Differential effects of leptin on adiponectin expression with weight gain versus obesity. Int. J. Obes..

[60] Konrad D., Somwar R., Sweeney G., Yaworsky K., Hayashi M., Ramlal T., Klip A. (2001). The antihyperglycemic drug α-lipoic acid stimulates glucose uptake via both GLUT4 translocation and GLUT4 activation: Potential role of p38 mitogen-activated protein kinase in GLUT4 activation. Diabetes.

[61] Konrad D. (2005). Utilization of the insulin-signaling network in the metabolic actions of alpha-lipoic acid-reduction or oxidation?. Antioxid. Redox Signal..

[62] Shay K.P., Moreau R.F., Smith E.J., Smith A.R., Hagen T.M. (2009). Alpha-lipoic acid as a dietary supplement: Molecular mechanisms and therapeutic potential. Biochim. Biophys. Acta Gen. Subj..

[63] Packer L., Cadenas E. (2011). Lipoic acid: Energy metabolism and redox regulation of transcription and cell signaling. J. Clin. Biochem. Nutr..

[64] Gorąca A., Huk-Kolega H., Piechota A., Kleniewska P., Ciejka E., Skibska B. (2011). Lipoic acid—Biological activity and therapeutic potential. Pharmacol. Rep..

[65] Lee W.J., Eun H.K., Jong C.W., Kim M.S., Park J.Y., Lee K.U. (2005). Obesity: The role of hypothalamic AMP-activated protein kinase in body weight regulation. Int. J. Biochem. Cell Biol..

[66] Carrier B., Rideout T.C. (2013). Anti-Obesity and Lipid- Lowering Properties of Alpha- Lipoic Acid. J. Hum. Nutr. Food Sci..

[67] Frühbeck G., Catalán V., Rodríguez A., Gómez-Ambrosi J. (2018). Adiponectin-leptin ratio: A promising index to estimate adipose tissue dysfunction. Relation with obesity-associated cardiometabolic risk. Adipocyte.

[68] Frühbeck G., Catalán V., Rodríguez A., Ramírez B., Becerril S., Salvador J., Colina I., Gómez-Ambrosi J. (2019). Adiponectin-leptin ratio is a functional biomarker of adipose tissue inflammation. Nutrients.

[69] Ferreira Y.A.M., Kravchychyn A.C.P., Vicente S.D.C.F., Campos R.M.D.S., Tock L., Oyama L.M., Boldarine V.T., Masquio D.C.L., Dâmaso A.R. (2020). Influence of magnitude of weight loss on Adipo/lep ratio in adolescents with obesity undergoing multicomponent therapy. Cytokine.

[70] Horgan S., Watson C., Glezeva N., Baugh J. (2014). Murine models of diastolic dysfunction and heart failure with preserved ejection fraction. J. Card. Fail..

[71] Conceição G., Heinonen I., Lourenço A.P., Duncker D.J., Falcão-Pires I. (2016). Animal models of heart failure with preserved ejection fraction. Neth. Heart J..

[72] Riehle C., Bauersachs J. (2019). Small animal models of heart failure. Cardiovasc. Res..

[73] Valero-Muñoz M., Backman W., Sam F. (2017). Murine Models of Heart Failure With Preserved Ejection Fraction: A “Fishing Expedition”. JACC Basic Transl. Sci..

[74] Packer M., Kitzman D.W. (2018). Obesity-Related Heart Failure With a Preserved Ejection Fraction: The Mechanistic Rationale for Combining Inhibitors of Aldosterone, Neprilysin, and Sodium-Glucose Cotransporter-2. JACC Heart Fail..

[75] Schiattarella G.G., Altamirano F., Tong D., French K.M., Villalobos E., Kim S.Y., Luo X., Jiang N., May H.I., Wang Z.V. (2019). Nitrosative stress drives heart failure with preserved ejection fraction. Nature.

[76] Shah A.M., Shah S.J., Anand I.S., Sweitzer N.K., O’Meara E., Heitner J.F., Sopko G., Li G., Assmann S.F., McKinlay S.M. (2014). Cardiac structure and function in heart failure with preserved ejection fraction: Baseline findings from the echocardiographic study of the treatment of preserved cardiac function heart failure with an aldosterone antagonist trial. Circ. Heart Fail..

[77] Tuseth N., Cramariuc D., Rieck Å.E., Wachtell K., Gerdts E. (2010). Asymmetric septal hypertrophy—A marker of hypertension in aortic stenosis (a SEAS substudy). Blood Press..

[78] Kim H., Yoon H.J., Park H.S., Cho Y.K., Nam C.W., Hur S.H., Kim Y.N., Kim K.B. (2011). Usefulness of tissue doppler imaging-myocardial performance index in the evaluation of diastolic dysfunction and heart failure with preserved ejection fraction. Clin. Cardiol..

[79] Dugo C., Rigolli M., Rossi A., Whalley G.A. (2016). Assessment and impact of diastolic function by echocardiography in elderly patients. J. Geriatr. Cardiol..

[80] Sun F., Huang Y., Li L., Wang Y., Zhuang P., Zhang Y. (2019). PKA/β2-AR-Gs/Gi signaling pathway is associated with anti-inflammatory and pro-apoptotic effects of Fuzi and Banxia combination on rats subjected to pressure overload. J. Ethnopharmacol..

[81] Wang T.J., Larson M.G., Levy D., Benjamin E.J., Leip E.P., Wilson P.W.F., Vasan R.S. (2004). Impact of Obesity on Plasma Natriuretic Peptide Levels. Circulation.

[82] Oh A., Okazaki R., Sam F., Valero-Muñoz M. (2019). Heart Failure With Preserved Ejection Fraction and Adipose Tissue: A Story of Two Tales. Front. Cardiovasc. Med..

[83] Nauta J.F., Hummel Y.M., Tromp J., Ouwerkerk W., van der Meer P., Jin X., Lam C.S.P., Bax J.J., Metra M., Samani N.J. (2020). Concentric vs. eccentric remodelling in heart failure with reduced ejection fraction: Clinical characteristics, pathophysiology and response to treatment. Eur. J. Heart Fail..

[84] Standeven K.F., Hess K., Carter A.M., Rice G.I., Cordell P.A., Balmforth A.J., Lu B., Scott D.J., Turner A.J., Hooper N.M. (2011). Neprilysin, obesity and the metabolic syndrome. Int. J. Obes..

[85] Wu C.-K., Lee J.-K., Chiang F.-T., Yang C.-H., Huang S.-W., Hwang J.-J., Lin J.-L., Tseng C.-D., Chen J.-J., Tsai C.-T. (2011). Plasma levels of tumor necrosis factor-α and interleukin-6 are associated with diastolic heart failure through downregulation of sarcoplasmic reticulum Ca2+ ATPase. Crit. Care Med..

[86] Keck M., Flamant M., Mougenot N., Favier S., Atassi F., Barbier C., Nadaud S., Lompré A.M., Hulot J.S., Pavoine C. (2019). Cardiac inflammatory CD11b/c cells exert a protective role in hypertrophied cardiomyocyte by promoting TNFR 2- and Orai3- dependent signaling. Sci. Rep..

[87] Markousis-Mavrogenis G., Tromp J., Ouwerkerk W., Devalaraja M., Anker S.D., Cleland J.G., Dickstein K., Filippatos G.S., van der Harst P., Lang C.C. (2019). The clinical significance of interleukin-6 in heart failure: Results from the BIOSTAT-CHF study. Eur. J. Heart Fail..

[88] Fontes J.A., Rose N.R., Čiháková D. (2015). The varying faces of IL-6: From cardiac protection to cardiac failure. Cytokine.

[89] Golbidi S., Badran M., Laher I. (2011). Diabetes and alpha lipoic acid. Front. Pharmacol..

[90] Ghibu S., Richard C., Delemasure S., Vergely C., Mogosan C., Muresan A. (2008). An endogenous dithiol with antioxidant properties: Alpha-lipoic acid, potential uses in cardiovascular diseases. Ann. Cardiol. Angeiol..

[91] Aimo A., Castiglione V., Borrelli C., Saccaro L.F., Franzini M., Masi S., Emdin M., Giannoni A. (2020). Oxidative stress and inflammation in the evolution of heart failure: From pathophysiology to therapeutic strategies. Eur. J. Prev. Cardiol..

[92] Münzel T., Gori T., Keaney J.F., Maack C., Daiber A. (2015). Pathophysiological role of oxidative stress in systolic and diastolic heart failure and its therapeutic implications. Eur. Heart J..

[93] van der Pol A., van Gilst W.H., Voors A.A., van der Meer P. (2019). Treating oxidative stress in heart failure: Past, present and future. Eur. J. Heart Fail..

[94] Franssen C., Chen S., Hamdani N., Paulus W.J. (2016). From comorbidities to heart failure with preserved ejection fraction: A story of oxidative stress. Heart.

[95] Stasch J.P., Schmidt P.M., Nedvetsky P.I., Nedvetskaya T.Y., Arun Kumar H.S., Meurer S., Deile M., Taye A., Knorr A., Lapp H. (2006). Targeting the heme-oxidized nitric oxide receptor for selective vasodilatation of diseased blood vessels. J. Clin. Investig..

[96] Ghibu S., Richard C., Vergely C., Zeller M., Cottin Y., Rochette L. (2009). Antioxidant properties of an endogenous thiol: Alpha-lipoic acid, useful in the prevention of cardiovascular diseases. J. Cardiovasc. Pharmacol..

[97] Kohen R., Nyska A. (2002). Oxidation of biological systems: Oxidative stress phenomena, antioxidants, redox reactions, and methods for their quantification. Toxicol. Pathol..

[98] Wollin S.D., Jones P.J.H. (2003). α-Lipoic Acid and Cardiovascular Disease. J. Nutr..

